# Nature-Inspired Chiral Structures: Fabrication Methods and Multifaceted Applications

**DOI:** 10.3390/biomimetics8070527

**Published:** 2023-11-06

**Authors:** Da-Seul Kim, Myounggun Kim, Soonmin Seo, Ju-Hyung Kim

**Affiliations:** 1 Department of Energy Systems Research, Ajou University, Suwon 16499, Republic of Koreakmk1491@ajou.ac.kr (M.K.); 2 Department of Chemical Engineering, Ajou University, Suwon 16499, Republic of Korea; 3 Department of Bionano Technology, Gachon University, Seongnam 13120, Republic of Korea

**Keywords:** chirality, enantiomers, mirror images, circular polarization

## Abstract

Diverse chiral structures observed in nature find applications across various domains, including engineering, chemistry, and medicine. Particularly notable is the optical activity inherent in chiral structures, which has emerged prominently in the field of optics. This phenomenon has led to a wide range of applications, encompassing optical components, catalysts, sensors, and therapeutic interventions. This review summarizes the imitations and applications of naturally occurring chiral structures. Methods for replicating chiral architectures found in nature have evolved with specific research goals. This review primarily focuses on a top-down approach and provides a summary of recent research advancements. In the latter part of this review, we will engage in discussions regarding the diverse array of applications resulting from imitating chiral structures, from the optical activity in photonic crystals to applications spanning light-emitting devices. Furthermore, we will delve into the applications of biorecognition and therapeutic methodologies, comprehensively examining and deliberating upon the multifaceted utility of chiral structures.

## 1. Introduction

The diversity of structures found in nature serves as a significant source of inspiration for contemporary research endeavors. Over millions of years, evolution and selective processes have created these structures with optimal designs and functionalities, continually offering researchers novel ideas and unexplored avenues. In particular, innovative solutions are being discovered across diverse fields, such as nanotechnology, materials engineering, medicine, and energy storage [1]. Amidst this spectrum of influence, research on chiral structures found in nature is also thriving. Chiral structures possess mirror-image forms in nature, exhibiting asymmetric characteristics akin to left and right hands. These structures exhibit unique physical, chemical, and optical properties, leading to their active exploration in various studies, including their application in nanoscale semiconductors and displays [2].

Chiral structures observed in nature come in a variety of shapes and sizes, starting from unique chemical structures identical to the helicene [3] and lanthanide complexes [4], and can be clearly seen in DNA [5], amino acids [6], snail shells [7], beetle cuticles [8,9], and even spiral galaxies [10]. Researching these natural models enables the design of novel chiral structures tailored for diverse applications. Researching these chiral models enables the design of novel chiral structures tailored for diverse applications. Foremost among these applications are photonics such as photonic crystal [11], circularly polarized luminescence dye [12], sensors for investigation of selective circularly polarized light [13], catalysis for selective activation and treatment [14], and biomedical therapy [15]. The unique properties of chiral structures are more evident not only in biological and chemical properties but also in optical and electromagnetic properties. The chiral electromagnetic properties of chiral structures, such as electrical magneto-chiral anisotropy dependent on external magnetic fields, current, and the handedness of chiral conductors [16], as well as chirality-induced spin selectivity influenced by handedness, are garnering significant attention [17]. These properties can be applied and utilized in the fields of spintronics and quantum computing.

In this review, we will focus on chiroptical properties, which are the optical characteristics of chiral materials. Optical activity via the interaction of chiral structures with light refers to the ability of chiral structures to rotate the vibration direction of light passing through them and convert linear polarization to circular polarization [18]. Circularly polarized light exhibits a rotating polarization state with a constant magnitude perpendicular to the direction of the electromagnetic field. The clockwise rotation of light from the light source is referred to as right-handed circular polarization (RCP), while the counterclockwise rotation is termed left-handed circular polarization (LCP), denoted as dextrorotatory (+) and levorotatory (−), respectively [19]. Exploiting this light rotation property allows for the analysis and utilization of chiral structures’ properties and optical changes. These optical rotation properties can be employed to interpret and utilize the inherent structure and optical activity of chiral materials. To achieve this objective, two analysis methods entail optical rotatory dispersion (ORD), which measures how much chiral molecules rotate plane-polarized light based on the wavelength, and circular dichroism (CD), which assesses the difference in light absorption with respect to left or right circularly polarized light [20,21,22]. Furthermore, various chiroptical properties can be employed to design new functional molecules and structures by optically manipulating chiral structures.

These studies and applications of such structures play a crucial role in developing technologies that manipulate and polarize light, efficient sensor technologies utilizing circularly polarized light, and innovative solutions through medical treatments and reactions. Owing to their fascinating properties, chiral structures are currently attracting significant attention and active research efforts.

In nature, observable chiral structures can be imitated via various approaches [23], and the choice of created chiral structures and materials is driven by applications of chirality, desired properties, as well as the utilization of fabrication or synthesis techniques. Taking these factors into consideration, the selection and design of constituents within chiral structures are vital, and experimental and simulation studies are conducted in tandem to determine the optimal materials and structures depending on the specific application. The approaches for crafting chiral structures are broadly categorized into both bottom-up and top-down methods. Bottom-up approaches utilize molecular chirality or intermolecular interactions to form complex and sophisticated structures, ranging from zero-dimensional structures such as chiral carbon dots [24] and chiral nanoparticles (NPs) to three-dimensional structures formed by supramolecular assemblies [25,26], primarily via molecular self-assembly and stereochemical manipulation [27,28]. This approach allows for precise control at the molecular level, thereby enabling the realization of various chiral structures and functionalities. In contrast, the top-down approach employs processes like lithography to precisely create chiral structures [29]. This method shapes material forms at the nanoscale using light or particle beams to generate desired chiral patterns [30]. This approach yields various chiral structures, allowing control over optical and chemical properties [31]. In the introduction to the emulation approaches in this review, the emphasis will be placed on emulating and fabricating geometric chiral structures through structuring metals or polymers in conjunction with the bottom-up approach. The fabrication methods showcased in this review open possibilities for diverse chiral structure creation.

These diverse imitations of chiral structures possess immense potential due to their unique optical properties. This has led to active research across multiple fronts. This review aims to delve into naturally occurring chiral structures, their fabrication methods, and applications spanning various domains.

## 2. Chiral Structures Found in Nature

Chiral structures, akin to the uniqueness of human fingerprints, are also discovered throughout nature across various life forms. These structures possess directionality resulting from rotation around a central axis, and the diverse characteristics that emerge from this rotation have inspired numerous studies today. Among the prominent chiral structures found in nature is the chiral nematic arrangement, also known as the cholesteric structure, found in liquid crystals. This structure, alternatively referred to as the Bouligand structure, is prevalent in nature, ranging from microscopic to macroscopic structures, including DNA. This Bouligand structure is not only present in the human body but also commonly observed in various organisms, including insects and plants.

**Figure 1:**
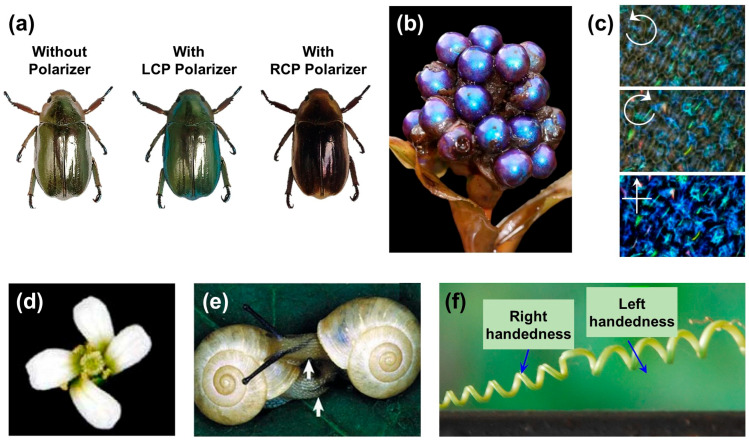
Bioentities with chiral architectures. (**a**) Reflection images of C. gloriosa in circularly polarized light. The photos were taken using LCP and RCP polarizers installed on the camera. Reproduced under the terms of the CC-BY 4.0 license [32]. Copyright 2018, Springer Nature. (**b**) Photograph of an alcohol-preserved specimen of *Pollia condensata* collected in 1974. (**c**) Polarization reflection images of *Pollia condensata* analyzed through a polarization filter. The arrows indicate the direction of polarization. Adapted from [34], based on free access. (**d**) Photograph of the petals of spiral2 rotated counterclockwise. Reproduced under the terms of the CC-BY 4.0 license [35]. Copyright 2020, MDPI. (**e**) Photograph of snails with different shell handedness. Reprinted from [36] with permission from Springer Nature. (**f**) Photograph of tendril helices with handles in both handedness. Reprinted from [37] with permission from Springer Nature.

One specific example of a distinctive insect displaying remarkable properties due to such structures is the scarab beetle (Figure 1a). This insect forms a Bragg band covering a wide spectral range (340 to 1000 nm) and exhibits LCP reflection [8,32]. This phenomenon arises from the lamellar structure of chitin in the wing, which leads to an arrangement akin to cholesteric liquid crystals. This unique appearance holds potential applications in diverse optical components, including structural colors. While most species similar to the scarab beetle reflect LCP, *Chrysina resplendens* exhibits RCP due to distinct birefringence, adding to its intriguing attributes [33]. These facts contribute to understanding the characteristics of chiral structures and have sparked heightened interest in the field of optical and optoelectronic device research involving these insects.

Such Bouligand structures can also be found in plants. The *Pollia condensata*, native to Africa, exhibits a metallic color that maintains vibrancy over time, as shown in Figure 1b. This phenomenon confirms that *Pollia condensata*’s color stems from structural colors, a result of the cholesteric arrangement of cellulose layers. Furthermore, this fruit can reflect both LCP and RCP. This polarization behavior can be classified using polarizing filters (Figure 1c), demonstrating that the color manifests polarization effects due to the cholesteric arrangement [34]. These findings extend to the genetic evolution that allows organisms to thrive in nature. Moreover, optical phenomena arising from cholesteric structures can also be observed in other organisms, such as the Mantis shrimp [38,39] and the Sheep crab [40]. Additionally, in plants, one can observe the asymmetrically rotated structures of flower petals. Most spiraling plants tend to wind around their supporting structures in a right-handed spiral. However, the wild Arabidopsis thaliana grows symmetrically without twisting its petals during normal development [35]. Nevertheless, genetic mutations leading to helical growth can cause the entire petal to exhibit a left-handed spiral (Figure 1d), similarly observed in snails and marine seashells [36,41,42]. Snails are often determined by maternal genes for their shell coiling. Due to the challenges in reproducing snails with anti-rotational chirality, like the one shown in Figure 1e, individuals of the same species typically exhibit chirality in a consistent direction.

In the natural realm, such chiral structures can also manifest even when a species exhibits both left-handed and right-handed morphologies. As shown in Figure 1f, in Towel Gourd tendrils, the direction of helical growth can be both left-handed and right-handed configurations, attributable to genetic variations within the plant population [37]. Additionally, a similar example is found in goat horns [43], where a non-superimposable symmetric chiral structure is evident. The array of chiral structures witnessed in the natural realm highlights the fascinating interplay of genetics and form, enriching our understanding of life’s intricate design.

## 3. Fabrication of Structures Inspired by Nature

The diverse chiral structures found in nature have provided significant inspiration and have been replicated in various forms. These structures share the common characteristic of rotation around a specific axis. In this section, we aim to classify natural chiral structures into three categories and review the major efforts to fabricate them artificially.

### 3.1. Bouligand Structures

**Figure 2:**
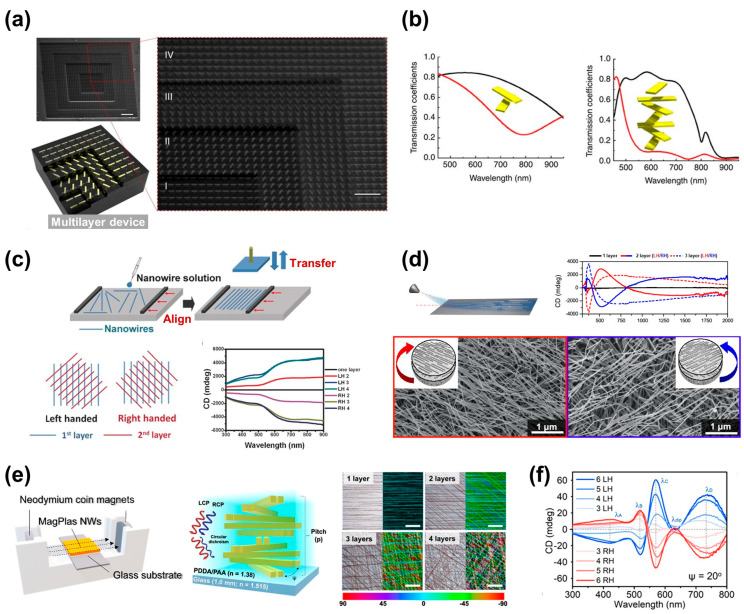
Fabrication method of Bouligand structures via stacking. (**a**) SEM images of multilayers with stacked Au nanorods and device illustration. (**b**) Frequency response variation with rotated and layered nanorods. Wider bandwidth was observed for 7-layered structure compared to 2-layered structure. The black and red lines represent the frequency response variation of RCP and LCP, respectively. Reprinted from [29] with permission from Springer Nature. (**c**) Representation of nano-wire alignment method using Langmuir–Blodgett technique and layered chiral film with opposite handedness. CD spectra with respect to layered structures are also shown. Reprinted from [50] with permission from John Wiley and Sons. (**d**) (**Top**) Illustration of nanowire alignment via spray deposition technique and optical activity graphs of chirally aligned and stacked structures. (**Bottom**) SEM images of chiral Au wires stacked at 60° rotation. Reprinted with permission from [52]. Copyright 2021 American Chemical Society. (**e**) Schematic of Au@Fe_x_O_y_ nanowire assembly via external magnetic field and chiral structure, along with optical microscopy images of fabricated bilayered structure. Color differentiated based on orientation. (**f**) CD spectra corresponding to numbers of nanowire layers. Reprinted with permission from [52]. Copyright 2022 American Chemical Society.

Extensively researched Bouligand structures merit considerable attention. In recent years, advancements in nanofabrication via both bottom-up and top-down approaches have rapidly developed chiral nanomaterials. Notably, these structures are fabricated through various methods, with bottom-up approaches such as the self-assembly of nano cellulose being extensively explored. Cellulose nanocrystal, a natural material, forms Bouligand structures through self-assembly via vapor methods [44]. This microstructure, akin to cholesteric liquid crystals, constitutes chiral molecules with spiral pitches used to selectively filter circular polarizations based on Bragg reflection [45,46]. This structure exhibits varying colors or chiroptical responses depending on the half-pitch while showcasing diverse characteristics based on length and synthesis methods [47,48]. Materials like cellulose with such attributes can also be combined with top-down approaches (e.g., soft lithography), designing structures that align with Bragg’s theory [49]. Y. Zhao et al. demonstrated the fabrication of metallic nanorods into multi-layered structures with controlled twist angles and well-ordered nanorod stacking structures using spacers (Figure 2a) [29]. The computational and experimental results exhibited strong chiroptical effects for RCP and LCP lights due to plasmon coupling as the metallic nanostructures were stacked (at θ = 60°). As more layers were added, the bandwidth gradually broadened in a step-like manner, with seven layers exhibiting chiroptical response characteristics spanning the entire visible range (Figure 2b). This confirmed that the optical phenomenon of original uniaxial materials can be produced using layer-by-layer stacking to resemble the optical behavior of conventional cholesteric systems. To address this issue, J. Lv et al. utilized Langmuir–Blodgett technology to align dispersed nanowires and rotated them at appropriate angles to demonstrate cholesteric arrays layer-by-layer, as depicted in Figure 2c [50]. The aligned nanowires showed well-oriented structures with deviations of less than 10° in over 90% of cases. Depending on the layers rotated at a 45° angle, this structure exhibited varying uniaxial optical phenomena. In the case of three-layer stacking, it revealed high CD values across a broad bandwidth. Similarly, using a combination of grazing incidence spraying (GIS) methods to facilitate easy layering and highly oriented nanowires, layers stacked at a 60° angle displayed varying CD values across a wide range (Figure 2d) [51]. At the upper part of the stacked structure, it achieved a high CD value of 4000 mdeg (at 380 nm), and the extinction of the metasurface was observed as the number of layers increased.

In addition, to enhance the precision of nanowire alignment, ongoing research has delved into methodologies that leverage external stimuli for orchestrated arrangements. For instance, in the research conducted by H.-Q. Nguyen et al., gold (Au) nanowires sheathed with magnetic oxide nanoparticles underwent alignment along a prescribed axis via the application of a magnetic field [52]. Figure 2e illustrates a schematic of structures and chiral arrangements for nanowire assembly. Aligned and stacked nanowires were observed to be oriented and layered in a consistent direction, and these aligned nanowires were layered to exhibit distinctive CD responses under varying circularly polarized light conditions (Figure 2f). The layers of nanowires, systematically rotated at consistent angles using the magnetic bar, evinced robust chiroptical response characteristics. Notably, as the number of stacked layers increased, this phenomenon intensified. These effects manifested across diverse uniaxial optical phenomena observed at discrete spectral dip positions (420, 512, 565, and 715 nm). At a wavelength of 614 nm, the relative anisotropy ratio demonstrated a pronounced chiral effect with an average magnitude of 0.72. This was in stark contrast to the uniaxial CD exhibited in the absence of such layered alignment. These advancements have markedly streamlined the process of stacking layers based on the chosen nanowire alignment technique and the specific angle of rotation. Furthermore, by exerting control over the particle concentration within the aqueous colloid solution, alongside the size of the nanowires, it becomes feasible to modulate the density of aligned nanowires. This, in turn, facilitates the creation of structures characterized by a spectrum of iridescent hues. Such top-down methodologies are forging novel avenues of technological exploration within the realm of crafting chiral structures employing nanomaterials.

**Figure 3:**
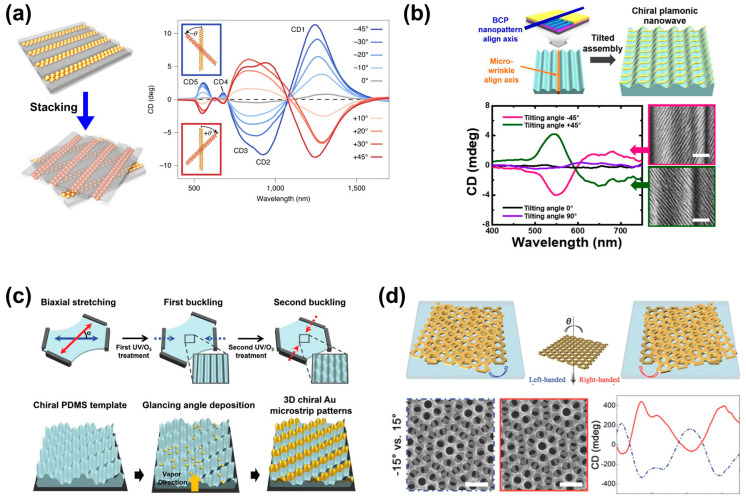
(**a**) Schematic diagram of chiral structure fabrication method using stacking of aligned gold nanoparticles with templates and optical activity analysis graphs varying with different stacking angles. Reproduced from [53] with permission from Springer Nature. (**b**) (**Top**) Schematic diagram of chiral structure fabrication through micro-wrinkled template and nano-pattern stacking. (**Bottom**) CD spectra with various inclination angles. (Inset: SEM images of nano-pattern transferred with +45° and −45° angles. The scale bar represents 200 nm.) Reprinted with permission from [54]. Copyright 2021 American Chemical Society. (**c**) Schematic diagram of a two-step buckling process for 3D chiral template fabrication. Fabrication process of Au microstrip pattern using GLAD deposition of Au layers. Inclination angle is 10°. Reprinted from [55] with permission from John Wiley and Sons. (**d**) (**Top**) Overview of moiré chiral structure fabrication using two Au membrane films. (**Bottom**) SEM images and CD spectra of in-plane rotated moiré chiral structure at 15° angle. Reprinted from [56] with permission from John Wiley and Sons.

Further advancement in the fabrication of intricate chiral Bouligand structures has been achieved using template-assisted techniques. Instead of controlling the orientation of wire-shaped materials, these techniques employ templates to arrange nanoparticles into arrays and adjust the template’s rotation angle to create mirror-imaged stacked structures. This approach goes beyond the limitations of material shapes with easy orientation, enabling a wider range of materials for chiral structure fabrication by controlling particles. These template-assisted techniques offer ease in modulating desired CD characteristics, encompassing sign, magnitude, and spectral position [53]. As depicted in Figure 3a, the structure was created by stacking metal nanoparticles in various angles atop a template composed of colloidal assemblies of nanoparticles, enabling complete and reversible post-modulation within the visible to near-infrared (NIR) range. This fabrication method, accommodating re-stacking, allows for the modulation of CD by adjusting particle arrangements and geometric configurations.

In this context, further research has been conducted using patterned templates to stack metal patterns, leading to the fabrication of 2D or 3D chiral structures. Primarily, by layering metal patterns onto patterned templates, distinct mirror-image chiral structures are crafted. Taking inspiration from crossed fingers, flexible templates are patterned through buckling. Uniaxial fine wrinkles in the template play the role of anisotropic objects [54]. Aligned block copolymer films are positioned at specific angles (±45°) and fabricated over large areas via shear rolling processes (Figure 3b). These nanopatterns exhibit chiroptical characteristics. Similarly, studies have progressed using strain to create 3D flexible templates onto which metal layers were adhered via physical vapor deposition [55]. As shown in Figure 3c, the 3D flexible templates fabricated via two cycles of biaxial stretching were able to realize 3D chiral Au microstrip patterns via e-beam evaporation. These enantiomeric (i.e., mirror image) structures demonstrated transmittance signals resembling those of computational simulations and allowed the production of 3D chiral structures without intricate lithography. Recently, research has also explored the stacking of nanopatterns without spacers to enhance strong chiroptical responses by reducing periodicity and amplifying localized plasmonic effects. Figure 3d indicates that patterned metal films are rotated at specific angles, producing chiral structures through moiré patterns [56]. These studies offer significant advantages as response characteristics can be modulated based on rotation angles and pattern periodicity. The absence of spacers between stacked layers leads to potent chiroptical responses due to localized plasmonic effects, garnering considerable attention.

### 3.2. Planar Structures

**Figure 4:**
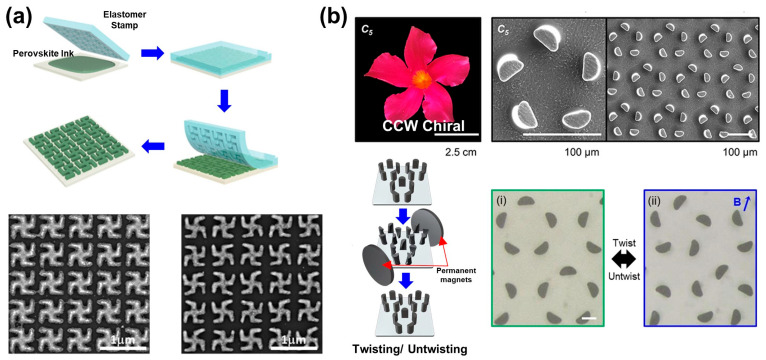
(**a**) Schematic representation of patterning process of perovskite nanocrystals via self-assembly, and SEM images of 2D chiral meta-surface. They possess structures of enantiomorphs of each other. Adapted from [57], based on open access. Copyright 2023, John Wiley and Sons. (**b**) (**Top**) SEM images of micro-pillar array inspired by asymmetric flower petals of Mandevilla. (**Bottom**) Schematic diagram of torsion operation induced by permanent magnets and optical microscope images of (i) untwisted and (ii) twisted micro-pillars due to an external magnetic field. Reprinted with permission from [58]. Copyright 2022 American Chemical Society.

In contrast to the previously mentioned stacked Bouligand structures, planar structures resembling the flowers of wild Arabidopsis thaliana (e.g., 2D propeller patterns) exhibit exceptional precision and control mainly using precise calculation and lithography techniques. Among various fabrication methods, soft lithography techniques, including nanoimprinting, offer relatively straightforward and cost-effective ways to fabricate patterns, making them favorable choices for creating planar structures. A large-scale chiral perovskite metasurface, produced using pre-patterned elastomeric stamps, can be fabricated into enantiomeric patterns (Figure 4a) [57]. In this work, two types of inks (emitted green/red) were employed to create chiral structures, enabling tunable circularly polarized luminescence. The resulting chiral metasurface exhibited up to 0.16 of photoluminescent dissymmetry factor, demonstrating the ease of controlling the optical properties of nanostructured inks. This technology has diverse applications and possesses high scalability. Further research has been conducted involving the synthesis of elastomer materials and magnetic substances to produce 3D chiral structures through magnetic fields [58].

Figure 4b shows a chiral structure created by artificially rotating the upper part via a magnetic field. This chiral selectivity is facilitated via magnetic torsion manipulation. Dynamic chiral transformation involves rapid transitions between magnetic twist modes by altering the magnetic field direction within a matter of seconds. This transition allows switching between left-handed and right-handed orientations. Each micro-filler undergoes chiral twisting operations at different angles based on magnetic field orientation and magnetic flux density. This approach stands apart from traditional permanently fixed chiral structures as it responds to magnetic influences and can change shape. It demonstrates the creation of deformable chiral structures, showcasing the ability to modify the configuration of chiral structures under the influence of a magnetic field.

### 3.3. Helical Structures

Advancing beyond planar structures, the realm of 3D helical structures holds transformative potential. These structures allow the creation of densely packed configurations, leading to enhanced chirality. Furthermore, they capitalize on spatial utilization to incorporate a diverse range of functionalities. However, the fabrication of 3D structures demands increasingly intricate techniques compared to 2D counterparts, necessitating persistent efforts to emulate them. To surmount this challenge, extensive research endeavors are in progress. Currently, studies are exploring techniques such as 3D printing and ion-beam lithography fabrication for producing these intricate structures. Nonetheless, these methods often come with high fabrication costs due to the complexity of generating precise patterns. As a response, research is intensifying its focus on discovering more economical and efficient fabrication methods.

**Figure 5:**
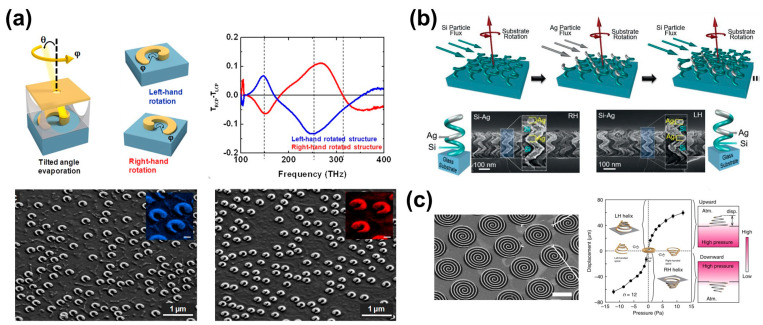
(**a**) (**Top**) Schematic diagram of a 3D chiral structure produced with different handles according to the parameter of GLAD Evaporation, which controls the angle of evaporation rotation. The structure is deposited via nanohole mask lithography fixed on a polymer scaffold. Polarization transmission difference of the 3D chiral structure produced at different rotation angles (φ) is also shown. Clear peaks are observed at three resonance positions. (**Bottom**) SEM images of the fabricated 3D chiral structure. Left-handed (blue) and right-handed (red) structures are confirmed based on the rotation angle. Reprinted with permission from [59]. Copyright 2013 American Chemical Society. (**b**) Schematic diagram of the fabrication method for a nano-helix array with Ag-Si heterostructures. SEM images show nano-helix structures with different orientations. Reprinted from [60] with permission from John Wiley and Sons. (**c**) SEM image of a spiral meta-surface with clockwise spiral rotation and graph analyzing displacement based on applied N_2_ gas pressure and direction (i.e., up/down). Adapted from [61], based on open access. Copyright 2015, Springer Nature.

One of the widely used approaches is applying the glancing angle deposition (GLAD) technique to create helical structures by adjusting deposition angles. Recent research reports the successful fabrication of large-scale 3D chiral structures in combination with GLAD and colloid nanohole lithography (Figure 5a) [59]. During the initial stages of metal evaporation, a significant amount of material deposits onto the substrate until the rotation speed gradually increases, controlling the amount of metal reaching the substrate. This process forms spiral-like lamp structures with diminishing diameters. The produced 3D helical Au structures were then cleaned to remove masks and supports, exhibiting a pronounced CD value of up to 13% in the frequency range of 100–400 THz, showcasing large-scale plasmonic chiral structures. Further attempts have been made to use the GLAD deposition technique to create helical structures with various compositions beyond single materials. U. Kilic et al. successfully fabricated a hybrid spiral structure comprising silver (Ag) and silicon (Si) [60]. By adjusting evaporation/rotation speeds through e-beam control, a helix structure with a 360 nm pitch was achieved (Figure 5b). This result implies the potential to fabricate chiral structures composed of diverse materials, not just single ones. The resulting products displayed enhanced chiral responses and also exhibited different Kuhn’s asymmetric coefficients (g-factor spectra) depending on their compositions.

In addition, recent studies have demonstrated the creation of 3D helical structures with varied handedness via deformation processes by applying pressure to previously fabricated 2D spiral structures (Figure 5c) [61]. The height of the structure varied corresponding to the intensity of N_2_ gas pressure applied in the upward and downward directions, which led to strong chiroptical response characteristics. This methodology allows for the facile fabrication of deformable helical structures without using the GLAD technique.

## 4. Applications of Chiral Structures

Chiral structures with enantiomeric configurations are optically active owing to their structural characteristics. Optical activity of such chiral structures and related phenomena can be graphically utilized in various fields of science and engineering, leading to a variety of applications. In this section, major applications of chiral structures are reviewed.

### 4.1. Photonics

**Figure 6:**
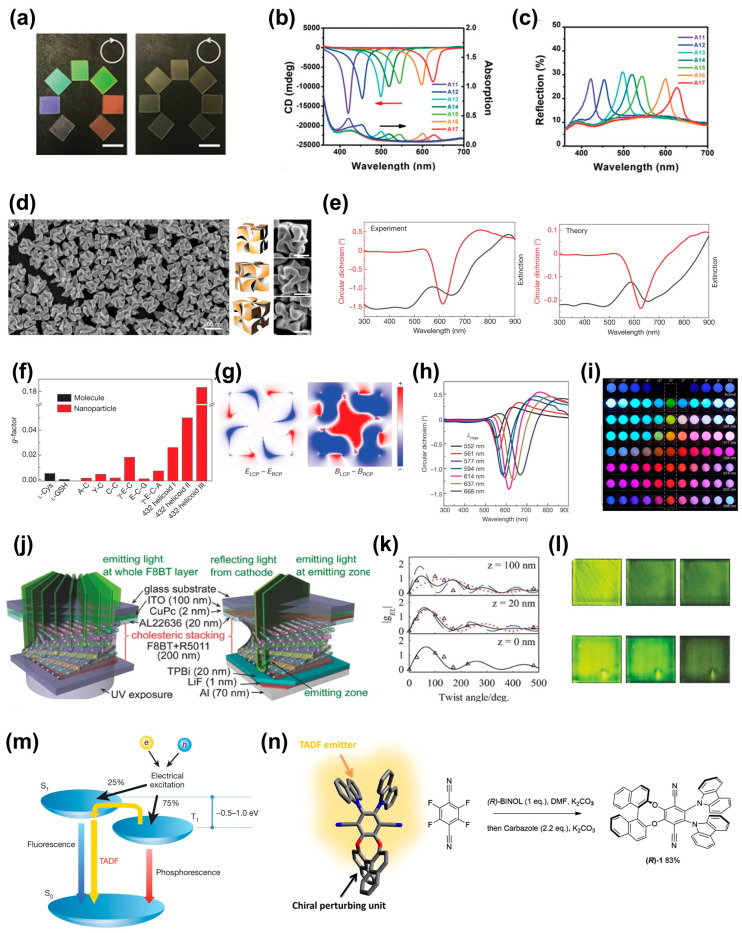
(**a**) Photographs of layer-by-layer transferred NiMoO4∙xH2O nanowire photonic crystals. Colors reflected through RCP (**left**) and LCP (**Right**). (**b**) CD and absorption spectra of the fabricated samples. (**c**) Reflection spectra. Reprinted from [11] with permission from John Wiley and Sons. (**d**) SEM images and illustrations of 432helicoid III from various angles. (**e**) Experimental and theoretical CD data (red lines) and extinction spectra (black lines) of 432helicoid III. (**f**) Comparison of dissymmetry g-factors with other NPs. (**g**) Theoretical calculation of the dependence of local electromagnetic fields on the handedness of circularly polarized light. (**h**) Wavelength-dependent CD spectra of 432helicoid III. (**i**) Polarized colors observed when various wavelengths are transmitted through 432helicoid III. Reprinted from [63] with permission from Springer Nature. (**j**) Schematic illustration of twisted stacking structure of F8BT molecules. (**k**) Variation of dissymmetry factor for EL with respect to the twist angle. Red and blue dotted lines indicate the g values for light propagation to anode and cathode, re-spectively. Black solid line represents the average of both dotted lines. (**l**) Images of pure F8BT samples and F8BT samples doped with 10wt% R5011 under light illumination. From left to right: no polarizer, right-handed circular polarizer, left-handed circular polarizer. Reprinted from [64] with permission from John Wiley and Sons. (**m**) Schematic illustration of TADF process. Reprinted from [65] with permission from Springer Nature. (**n**) Chiral structure of CPL-SOMs, and schematic image of chirality induction through the one-pot sequential synthesis. Reprinted with permission from [66]. Copyright 2016 American Chemical Society.

The prominent optical activity of chiral structures is harnessed for diverse optical components. In particular, the unique optical phenomena exhibited by chiral structures contribute to fields such as optics and displays [62]. As shown in Figure 6a, Bouligand structures fabricated using nanowires can play a role as tunable photonic crystals with vivid reflection colors [11]. In this work, the chiral photonic crystals, based on Bouligand structures, selectively reflected RCP and LCP lights corresponding to their rotating directions (clockwise and counterclockwise). The CD peaks exhibited full width at half maximum (FWHM) values of less than 30 nm and clearly showed reflection colors according to the pitches of the crystals (Figure 6b,c). Further research has also been conducted to confine similar structures in NPs. For this work, Au NPs were synthesized using cysteine or cysteine-based peptides [63]. By imparting chirality to these Au NPs, a material possessing excellent optical activity (designated 432helicoid III) was crafted (Figure 6d–h). The size of these NPs can be manipulated to control the degree of polarization of light, thereby modulating the polarization-resolved color of transmitted light (Figure 6i). These results are possibly applicable to filter-free displays.

The inspiration from stomatopod eyes has led to advancements in controlling polarization [67,68]. Stomatopods, by undergoing tortional rotation, exhibit enhanced circular polarization detection capabilities, allowing them to distinguish between ordinary and polarized lights within the visible range. This phenomenon can be artificially induced by creating a surface structure, which is known as an optical differential line metasurface. Chiral metasurfaces configured via multi-stacked layers enable the conversion between linearly polarized and circularly polarized lights, facilitating the production of circular polarizers with high extinction ratios and transmission efficiencies. These principles can also be applied to Stokes polarimetry measurements.

In addition, the optical characteristics of chiral structures are being extensively explored for the development and further refinement of display devices. One of the notable advancements in this realm is the development of circularly polarized organic light-emitting diodes (CP-OLEDs) (Figure 6j–l). The CP-OLEDs were demonstrated using twisted stacking structures, exhibiting high dissymmetry factors of 0.72 and 1.13 for photoluminescence (PL) and electroluminescence (EL), respectively. The dissymmetry factor represents the chiroptical response, which is influenced by retardation, the twisted angle of the emitting layer, and the degree of linear polarization of the emitted light [64]. In comparison with conventional OLEDs, circular polarizers are not required for the CP-OLEDs to emit circularly polarized light, of which the brightness efficiency can be significantly enhanced by the high dissymmetry factors (i.e., up to 60% in this work). These results suggest the great potential of CP-OLEDs, leading to excellent energy efficiencies and high-quality displays.

Another achievement involves the incorporation of chiral structures into the thermally activated delayed fluorescence (TADF) process originating from reverse inter-system crossing from triplet exciton to singlet exciton (Figure 6m) [65]. S. Feuillastre et al. reported that chiral photoluminescent small organic molecules (CPL-SOMs) were synthesized by combining TADF emitters and chiral structures (Figure 6n) [66]. This work could be achieved via a one-pot sequential synthesis method, introducing chirality to TADF molecules using 1,1′-bi-2-naphthol. By integrating the advantageous characteristics of both TADF emitters and chiral structures through the synthetic approach, CPL-SOMs were successfully engineered with high quantum efficiency reaching up to 74%. These results offer promising potential for further development of EL-based applications.

### 4.2. Catalysts

Inorganic supraparticles (SPs) are inorganic structures formed by assembling multiple NPs into functional modules [69]. These SPs exhibit characteristics similar to biological nanostructures in terms of size and shape. When forming nanostructures, they can range from simple shapes to intricate forms such as helices. SPs offer flexibility and modular engineering, allowing the incorporation of biological nanoscale structures [70]. The presence of chiral atomic surfaces and chiroptical features enables the induction of specific chemical reactions enantiospecifically, making them suitable for catalytic applications. Most SPs are created by combining a desired functional module with Au NPs, which is due to the unique properties of Au as a catalyst with high selectivity and stability.

**Figure 7:**
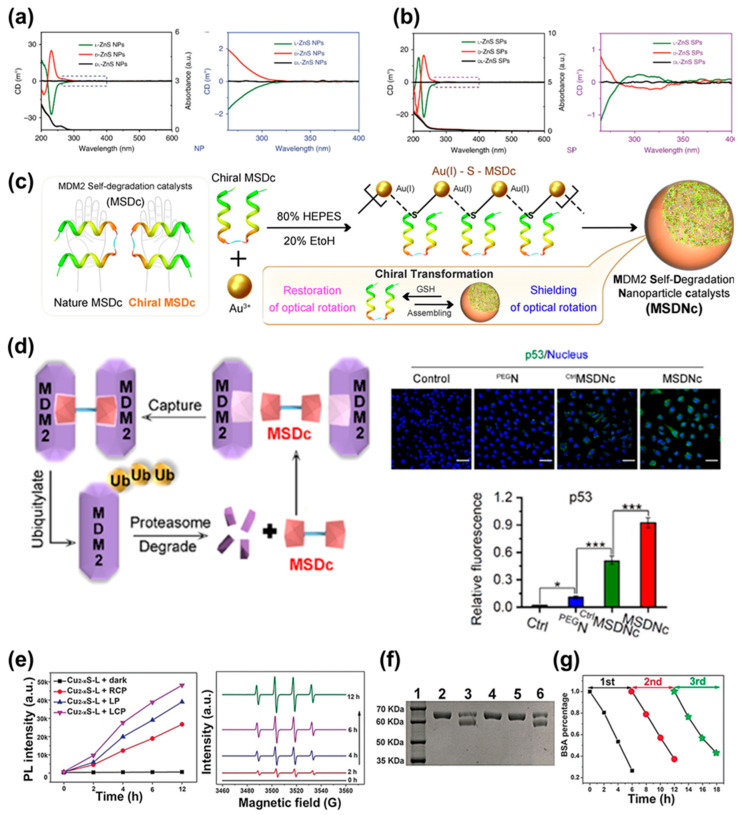
(**a**) CD and UV spectra of ZnS NPs. (**b**) CD and UV spectra of ZnS SPs including Au. Adapted from [71], based on open access. Copyright 2019, Springer Nature. (**c**) Schematic illustration of the structure and synthesis process of MSDNc. (**d**) Schematic illustration of the process of MSDNc degrading MDM2 and fluorescence images of p53 protein in cells. Quantitative comparison of p53 is also shown. * and *** indicate that p-values are less than 0.05 and 0.001, respectively. Reproduced under the terms of the CC-BY license [72]. Copyright 2023, Elsevier. (**e**) Generation of ROS from QDs under various light sources and detection of hydroxyl radical via electron paramagnetic resonance (EPR) under various light sources. (**f**) Results of SDS-PAGE analysis for BSA degradation under LCP irradiation (1: molecular marker, 2: BSA control, 3: BSA + QD, 4: BSA + QD + KI, 5: BSA + QD + ethanol, 6: BSA + QD + NaN_3_). (**g**) Recyclability assessment of QD for BSA cleavage. Reprinted from [73] with permission from John Wiley and Sons.

For instance, research has been conducted using ZnS-Au SPs as catalysts in photocatalytic enantioselective oxidation reactions of L- or D-Tyrosine (Tyr) [71]. By combining ZnS NPs and Au NPs, the catalytic performance in photoreactions was enhanced. Comparing the CD and ultra-violet (UV) spectra of ZnS NPs (Figure 7a) and ZnS SPs containing Au (Figure 7b) reveals distinct differences at a wavelength of 306nm, which corresponds to the PL peak when Tyr is oxidized to dityrosine (diTyr) in a photochemical reaction. This study showcases the potential of creating various SPs with diverse properties by combining different chiral nanoparticles. The integration of chiral properties into the assembly of inorganic nanostructures like SPs holds promise for catalysis, optical applications, and other fields, where the unique characteristics of these structures offer innovative ways to control and enhance various chemical and optical processes.

Another example involves the combination of chiral MSDc (murine double minute 2 (MDM2) self-degradation catalysts) with Au^3+^ ions to create self-assembled SPs with the effect of MSDc, termed MDM2 self-degradation nanoparticle catalysis (MSDNc) (Figure 7c) [72]. MDM2 is a protein that inhibits the function of p53, a protein responsible for inhibiting tumor growth. MSDNc is designed to induce the self-degradation of MDM2, promoting the growth of p53 and inhibiting tumor formation. To create chiral MSDNc, D-enantiomeric cysteine residues were introduced to the C-terminus of MSDc and assembled with Au^3+^ ions. The resulting MSDNc is more effective at inhibiting MDM2 compared to MSDc alone (Figure 7d).

Circularly polarized light can induce optical reactions and be used for catalytic reactions in biological systems. CdTe quantum dots (QD) can be employed for DNA cleavage (Figure 7e–g) [73]. CD analysis confirmed that LCP generates more reactive oxygen species (ROS) than RCP, indicating that specific nuclease mimetic activity can be achieved by controlling the ratio of light. This allows for the development of artificial endonucleases (enzymes that cleave DNA or RNA). Additionally, using chiral Cu_(2−x)_S QD created with L- and D-penicillamine, ROS can be utilized to promote the degradation of bovine serum albumin (BSA) (Figure 7f). L-Cu_(2−x)_S exhibited higher catalytic efficiency compared to D-Cu_(2−x)_S, inducing the decomposition of proteins. These examples show the intricate interplay between chiral properties and catalysis, leading to the development of novel materials and catalysts with applications in biomedicine, catalysis, and other fields.

### 4.3. Biosensors

Within cells, along with the role of catalysts, chiral structures offer the potential for successful sensing of external materials. Circularly polarized light can effectively sense various metal ions within live cells. Biosensors must have enantioselectivity to sense only the target material [74]. Research concerning DNAzymes has encountered challenges wherein the DNAzymes react with external metal ions or cleave substrates before reaching the desired intracellular locations [75]. To overcome these difficulties, studies are underway to combine DNAzymes with Au nanorods (NRs) or Au nanoshells (NSs) as a solution.

**Figure 8:**
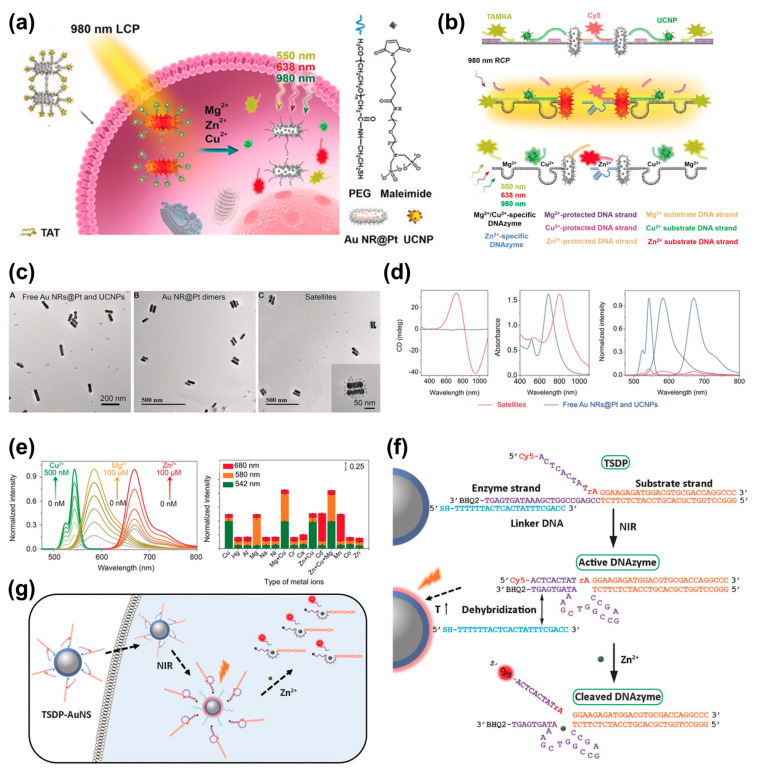
(**a**) Schematic illustration of Au@NR@PT dimer-UCNP satellites capable of detecting three metal ions. (**b**) Process of luminescent signal emission from Cy5, TAMRA, and UCNP by circularly polarized light. (**c**) TEM images of the mixture of Au NR@PT dimers and UCNPs, Au NR@Pt dimer, and combination of Au NR@Pt dimers and UCNPs as satellite assemblies. (**d**) CD, UV/vis absorption, and luminescence spectra of satellite assemblies compared with free Au NRs@Pt and UCNPs. (**e**) Normalized fluorescence intensity of satellite assemblies for Cu^2+^, Mg^2+^, and Zn^2+^. Selectivity of the structure under 980 nm RCP light. Reprinted from [76] with permission from John Wiley and Sons. (**f**,**g**) Process of DNA cleavage triggered by the activation of TSDP-AuNS by NIR light. Reprinted from [77] with permission from John Wiley and Sons.

Figure 8a shows that a chiral satellite nanoprobe was created by combining Au NR@Pt dimers with un-conversion nanoparticles (UCNPs) [76]. The surface of Au NR@Pt was modified with a thiolated Zn^2+^-specific DNAzyme and a thiolated Zn^2+^-protected DNA strand. Subsequently, Cu^2+^- and Mg^2+^-specific DNAzymes were introduced onto the dimer’s surface (Figure 8b). In this structure, circularly polarized light was emitted, and in the presence of Zn^2+^, Cu^2+^, and Mg^2+^ metal ions, the DNAzymes were activated, resulting in DNA sequence cleavage (Figure 8c–e). Under these conditions, cyanine (Cy5), tetramethylrhodamine (TAMRA), and UCNPs emit signals for each ion, allowing the detection of metal ions. These probes have excitation wavelengths of 638 nm, 550 nm, and 980 nm, respectively (Figure 8e).

Figure 8f depicts the hybridization of three-stranded DNAzyme precursor (TSDP) with Au NSs that occurs without undergoing cleavage, allowing it to reach the desired location. Subsequently, upon receiving near-infrared (NIR) light, the temperature increases, causing dehybridization at the target site and activating the cleavage process (Figure 8g) [77]. This leads to the emission of metal ions. By detecting these metal ions, it is possible to confirm whether the biomaterial has reached the intended location and whether the desired process has operated effectively. This approach could be utilized to detect various target substances within live cells. This approach holds potential for applications in cancer therapy or drug delivery systems, where drugs need to reach specific sites and perform targeted actions.

### 4.4. Therapies

As previously mentioned, the phenomenon of circularly polarized light induced by chiral structures can be applied to therapies and drug delivery systems [78,79]. In recent years, photodynamic therapy (PDT) and photothermal therapy (PTT) have garnered significant attention in the field of cancer treatment [80]. The fundamental principle of PDT involves delivering photosensitizers (PSs) to precise targets and then irradiating them with appropriate wavelengths of light, causing a photochemical reaction by the PSs and effectively eliminating tumors [81]. PTT works by absorbing NIR light and emitting heat to destroy cancer cells in the surrounding area [82]. However, these methods often impact healthy surrounding cells or face challenges in achieving desired drug quantities. To address these issues, the utilization of chiral nanostructures has gained prominence in the development of safe cancer treatment techniques that generate desired drug quantities specifically at targeted sites. This approach ensures precise and effective treatment, minimizing damage to healthy cells in the vicinity while concentrating on the desired areas.

**Figure 9:**
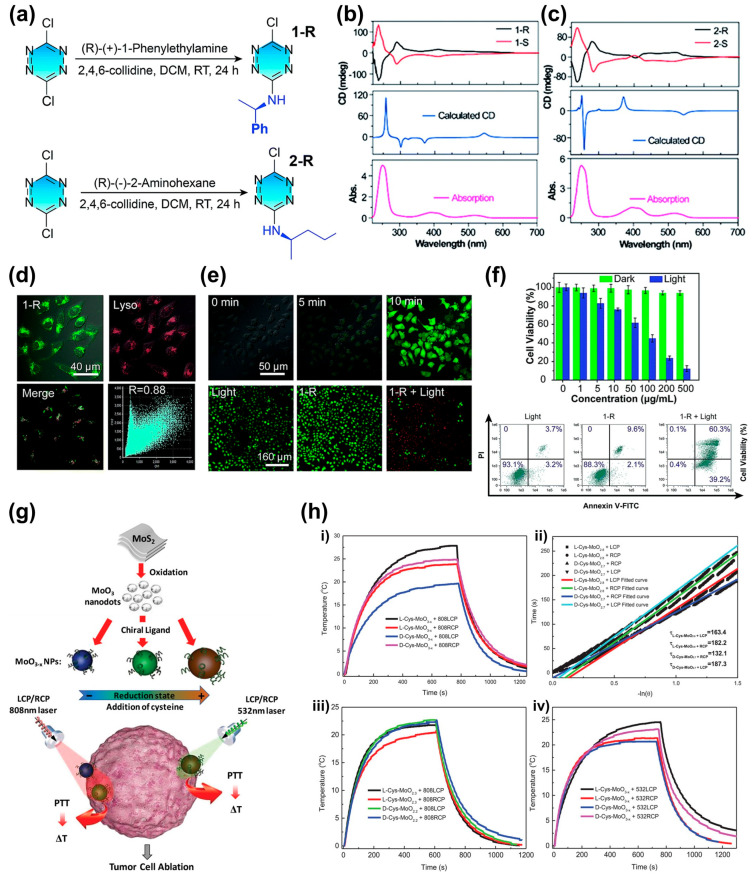
(**a**) Generation process and structural formula of 1-R and 2-R. (**b**,**c**) CD spectra of 1-R and 2-R. (**d**) Fluorescence images of **1**-R and lysosome-tracker (Lyso) in HeLa cells. (**e**) (**Top**) ROS generation from 1-R in HeLa cells. (**Bottom**) cell viability. Surviving cells are shown in green, while apoptotic cells are shown in red. (**f**) (**Top**) Cell viability under light illumination and without light illumination. (**Bottom**) Flow cytometry analysis. Reproduced from [83] with permission from the Royal Society of Chemistry. (**g**) Schematic illustration of Cys-MoO_3−x_ NPs. The wavelength range of the responsive spectrum varies according to the degree of reduction. (**h**) Temperature vs. time curves ((i) and (iii) under 808 nm LCP/RCP, and (iv) under 532 nm LCP/RCP). Plots in (ii) are fitting plots of (i), based on Roper’s method. Laser output for all samples is maintained at 1 W cm^−2^. Reprinted from [84] with permission from John Wiley and Sons.

A previous study examined the synthesis of tetrazine derivatives (designated 1 and 2) by substituting (R or S)-1-phenylethylamine and (R or S)-2-aminohexane onto a 3,6-dichlorotetrazine core (Figure 9a) [83]. This approach achieved efficient intersystem crossing, demonstrating the potential for PDT. Figure 9b–f shows the results of this study. Fluorescein isothiocyanate-labeled annexin V (annexin V-FITC) staining was used to distinguish between survival, necrosis, early apoptosis (damaged but not yet apoptotic), and late apoptosis (apoptotic) cells (Figure 9f). The results indicate that a significant portion of cells underwent apoptosis, demonstrating the efficacy of tetrazine derivative 1 in PDT by efficiently eliminating ROS.

In addition, Figure 9g depicts the application of chiral structures in PTT [84]. By controlling the absorption properties in the visible and NIR regions, substoichiometric molybdenum oxide MoO_(3−x)_ NPs were reduced from an oxygen-deficient state using chiral cysteine as both a reducing and capping agent. This process resulted in chiral plasmonic transitions in the NIR region and metal-to-ligand charge transfer (MLCT) chirality in the visible region. The three types of MoO_(3−x)_ NPs derived from this process demonstrated the potential for highly efficient tumor cell ablation in circularly polarized visible or NIR CPL (Figure 9h).

In summary, the use of circularly polarized light in PDT and PTT has gained significant attention in the field of cancer treatment and therapy techniques. In particular, ongoing research is exploring innovative approaches utilizing chiral nanostructures to enhance the effectiveness and safety of cancer treatment methods.

## 5. Conclusions

Research on bioinspired chiral structures found in nature has continued to evolve in various dimensions. In this review, we delve into the chiral structures of diverse organisms, ranging from insects to plants, and discuss the methods used to replicate these structures. Various fabrication techniques have been employed to mimic these structures, encompassing a wide range, including Bouligand structures, as well as planar and helical structures. Moreover, the potential for various applications via chiral structure utilization, driven by chiroptical response characteristics, underscores the potential for enhanced possibilities through bioinspired chiral structure emulation. The advantages of chiral structures, such as chiroptical and enantioselectivity, can be effectively applied in various fields. The main text introduces four primary types of applications. Firstly, in the field of photonics, the property of chiral structures to absorb and reflect light of specific wavelengths is being harnessed to develop more efficient displays, and other research is dedicated to the synthesis of CPL-SOMs combined with TADF. Secondly, in the catalysts field, various biocatalysts are under research using different SP modules, and DNA cleavage is being investigated with the use of QDs. Thirdly, in the biosensors field, chiral structures are employed to detect the presence and location of target materials within the live cells. This can enhance the stability of cancer treatments or drug delivery systems. Lastly, in the field of therapies, chiral structures capable of serving as both catalysts and sensors demonstrate potential for applications such as PDT and PTT. Thus, the emulation of chiral structures observed in nature is anticipated to pave the way for future innovations and advancements.

## Data Availability

Not applicable.
